# Research Gap in “Evaluating the Preparedness of Healthcare Providers for Prone Position CPR Across Jordan's Healthcare Sectors”

**DOI:** 10.1002/hsr2.71189

**Published:** 2025-08-27

**Authors:** Shahzaib

**Affiliations:** ^1^ Ayub Medical College Abbottabad Pakistan

To the Editor,

We read with interest the article by Al Hroub, “Evaluating the Preparedness of Healthcare Providers for Prone Position CPR Across Jordan's Healthcare Sectors.” The research points to an important and timely problem, providing useful baseline data regarding the preparedness of healthcare providers for prone position cardiopulmonary resuscitation (PPCPR) [1].

To point out some other limitations that can influence the generalizability and interpretation of the results. First, the sole use of self‐evaluation allows room for subjectivity and will not necessarily represent true clinical proficiency; external simulation‐based assessments have been demonstrated to be more accurate in quantifying readiness [2].

Second, the study does not assess the quality, content, or frequency of previous training variables consistently shown to have a deep effect on resuscitation performance. Without it, one cannot know if increased scores on the preparedness instrument are a reflection of good training or merely pre‐exposure.

Third, all participating hospitals were urban, tertiary‐level hospitals, which limits generalizability to rural or lower‐resource settings, where preparedness may be even lower [3]. In these environments, there may be less access to formal training, standardized orders, or simulation labs, and again, the preparedness gap widens. Extending the hospital sample to include a greater diversity of hospitals may help increase the external validity of the results.

Fourth, shift time, past clinical experience with PPCPR, and institutional policy were not controlled for each of these may be confounding factors that can influence levels of preparedness. Variability in exposure, workload, and protocol awareness between shifts and institutions may result in variable training outcomes.

Finally, the cross‐sectional design only sees a snapshot in time, and longitudinal follow‐up would be required to understand skill retention and the effect of training [4]. Short of following through over time, it is hard to measure whether gains in preparedness are lasting or temporary effects.

In spite of these constraints, the research offers a significant basis for the continued exploration of PPCPR preparedness. The inclusion of objective skill ratings, more extensive sampling, and longitudinal analysis would make future research more rigorous.

## 1 Author Contributions

**Shahzaib:** writing – original draft.

## Ethics Statement

The author has nothing to report.

## Consent

The author has nothing to report.

## 2 Transparency Statement

Author confirm that this Letter to the Editor is true, complete and open representation of my opinions and nothing critical has been left out, and any divergences from my intial purposes are entirely justified. Author attest that all procedures and sources are explained with the transparency and clarity typical for HSR standards of reproducibility and transparency, and that there are no conflicts of interest not disclosed.

## Data Availability

Data sharing is not applicable to this article as no data sets were generated or analyzed during the current study.

## References

[1] A. Al Hroub , S. Al‐Yatim , and M. Al‐Ruzzieh , “Evaluating the Preparedness of Healthcare Providers for Prone Position CPR Across Jordan's Healthcare Sectors: A Descriptive Cross‐Sectional Study,” Health Science Reports 8 (2025): e70955. 40606313 10.1002/hsr2.70955 PMC12215824

[2] H. W. Jeong , D. Ju , A. K. Lee , et al., “Effect of a Hybrid Team‐Based Advanced Cardiopulmonary Life Support Simulation Program for Clinical Nurses,” PLoS One 17, no. 12 (2022): e0278512. 36525410 10.1371/journal.pone.0278512 PMC9757587

[3] S. T. Veettil , M. S. Anodiyil , H. Khudadad , et al., “Knowledge, Attitude, and Proficiency of Healthcare Providers in Cardiopulmonary Resuscitation in a Public Primary Healthcare Setting in Qatar,” Frontiers in Cardiovascular Medicine 10 (2023): 1207918. 37534275 10.3389/fcvm.2023.1207918 PMC10390828

[4] A. Shrivastava and M. K. Singla , “Impact of Advanced Cardiac Life Support Training Program on the Outcome of Cardiopulmonary Resuscitation in a Tertiary Care Hospital,” Indian Journal of Critical Care Medicine 15, no. 4 (2011): 209–212. 22346031 10.4103/0972-5229.92070 PMC3271556

